# Histone Deacetylase Inhibitors Increase the Embryogenic Potential and Alter the Expression of Embryogenesis-Related and HDAC-Encoding Genes in Grapevine (*Vitis* *vinifera* L., cv. Mencía)

**DOI:** 10.3390/plants10061164

**Published:** 2021-06-08

**Authors:** Óscar Martínez, Verónica Arjones, María Victoria González, Manuel Rey

**Affiliations:** 1 Departamento de Biología Vegetal y Ciencia del Suelo, Universidad de Vigo, Campus Universitario, 36310 Vigo, Spain; oscarmtroncoso@gmail.com (Ó.M.); arjonesgonzalezveronica@gmail.com (V.A.); 2 Departamento de Biología Funcional, Universidad de Santiago de Compostela, Campus Sur, 15872 Santiago de Compostela, Spain; mvictoria.gonzalez@usc.es

**Keywords:** somatic embryogenesis, *Vitis* *vinifera*, gene expression, sodium butyrate, trichostatin A, *BABY BOOM*, *SERK2*, histone deacetylase-encoding genes

## Abstract

The low induction rates of somatic embryogenesis are one of the main limitations in its routine application in the grapevine (*Vitis vinifera* L.). The use of an induction medium containing histone deacetylase inhibitors (trichostatin A and, mainly, sodium butyrate) resulted in an improvement of the embryogenic responses in grapevine (cv. Mencía) cotyledonary and recently germinated somatic embryos. The relative expression of several grapevine genes related to embryogenic competence or encoding histone deacetylase enzymes was studied in cotyledonary somatic embryos that were cultured in the presence of 0.5 mM sodium butyrate. The results showed a significant overexpression of the *BBM* and *VvSERK2* genes after 24 h of culture, whereas the *VvWOX2* gene was underexpressed less in treated versus untreated explants. The results suggest that the inhibitor may trigger a molecular response related to an increase in embryogenic competence and changes in the expression of associated genes. The treatment with sodium butyrate also produced significant variations in the expression of several histone deacetylase enzyme-encoding genes. These results may enhance the possibility of obtaining somatic embryos, reducing the seasonal constraints associated with the use of floral explants in grapevines.

## 1. Introduction

Somatic embryogenesis is the physiological process by which somatic cells, under appropriate induction conditions, undergo a redifferentiation process and acquire embryogenic cell competences. Subsequently, these cells undergo a series of morphological and biochemical changes that culminate in the formation of somatic embryos and the generation of new plants [1]. Somatic embryogenesis is a powerful biotechnological tool that is particularly useful in species such as the grapevine. In this species, genetic improvement by traditional methods is inefficient due to factors such as severe inbreeding depression, a long-life cycle with late sexual maturity (which makes breeding tedious and time consuming), extreme heterozygosity, sex incompatibility (which makes the transfer of traits among *Vitis* species impossible due to their different numbers of chromosomes), and the difficult development of new cultivars due to consumer preferences for wines from specific cultivars [2].

The application of somatic embryogenesis to grapevines began in the late 1970s [2,3], and since then, the number of protocols available for different cultivars has increased exponentially [3]. Thus, the efficiency of the induction of somatic embryogenesis depends mainly on the interaction of the genotype, the culture medium, the explant used, and the degree of development of the explant itself [4,5,6].

The most successful explants for inducing somatic embryogenesis in grapevines are reproductive structures such as anthers [6], ovaries [6,7], stigmas [8], stamen filaments [9,10] or whole flowers [11]. In contrast, the acquisition of embryogenic competence using vegetative structures such as leaves, petioles, and nodal explants has been achieved on a few occasions and with low induction rates [2,3,12]. The practical implication of this phenomenon is that the establishment of new embryogenic crops is mostly restricted to the grapevine’s flowering period only, when the reproductive structures are available at the appropriate stage of development (just one week per year). For this reason, the establishment of a methodology to obtain somatic embryos from other grapevine tissues represents a key step in optimizing the use of somatic embryogenesis in this species [13].

However, although some of the genes involved in the acquisition of embryogenic competence have been identified, the exact mechanism that regulates the whole process is not clear, preventing the application of this technique on a routine basis. One of the alternatives to increase the embryogenic capacity of recalcitrant tissues is the modification of the expression of genes related to embryogenic competence, such as *BABY BOOM* (*BBM*), *Somatic Embryogenesis Receptor Kinase* (*SERK*), *Leafy Cotyledon* (*LEC*), or *Wuschel (WUS)-related homeobox* (*WOX*). The ectopic expression of the *SERK1* and the *BBM* genes has been shown to increase the efficiency of the initiation of somatic embryogenesis in *Arabidopsis* [14,15], while the overexpression of the *WUS* gene in different tissues of this species (roots, petioles, stems, and leaves) induces the formation of somatic embryos [16]. Furthermore, the pattern of expression of the *LEC1* and *LEC2* genes has been observed to be similar to that of *WUS* during the somatic embryogenesis of *Arabidopsis* [17], suggesting that the *LEC* genes are also involved in this process.

In grapevines, Schellenbaum et al. [18] characterized three *SERK* genes and analyzed the putative existence of *LEC* genes; they found a single sequence (*VvL1L*) showing the characteristic domains of the *Leafy cotyledon1*-*Like* (*L1L*) proteins of *Arabidopsis*. The *VvAP2-16* gene, belonging to the *apetala2* (*AP2*) family, has been identified as a putative *BBM* gene in grapevines [19]. A total of 12 *WOX* genes have been characterized in grapevines, which were named *VvWUS* and *VvWOXn*, according to their similarity with the sequences already described in *Arabidopsis* [20].

Recently, it was discovered that reversible changes in histone acetylation play an essential role in the regulation of gene expression during plant regeneration (recently reviewed by the authors in [21,22]). In general terms, the acetylation of histone lysine residues produces a relaxation of the chromatin structure, and this phenomenon is associated with increased gene activity [23,24]. In contrast, the elimination of these acetyl groups leads to a compaction of chromatin, often related to repression and gene silencing [25]. The balance between histone acetylation and deacetylation is controlled by the activity of histone acetyltransferases (HATs) and histone deacetyltransferases (HDACs), respectively [26]. The number of coding genes for these enzymes is high, and there are several gene families for each group of enzymes. Thus, two categories of HATs have been characterized according to their cellular distribution: type A, which is responsible for acetylation at the nuclear level, and type B, which catalyzes the acetylation of histone H4 in the cytoplasm. In the case of HDACs, the HD2 family (which is exclusive to plants), the sirtuin family (SIR2), and the RPD3/HDA1 superfamily have been described [27].

In grapevines, a total of 7 HAT enzyme-encoding genes and 13 HDAC-encoding genes have been identified [27]. Of these 13 HDAC genes, one belongs to the HD2 family and has been named HDT in grapevines; two belong to the sirtuin family and have been named SRT; and the remaining 10 are part of the RPD3/HDA1 superfamily and have been named HDA.

Treatment with HDAC inhibitors has been shown to increase histone acetylation [28] and influence many physiological processes [29]. Furthermore, these inhibitors have been shown to partially arrest the progression of germination, thus maintaining the embryogenic potential of *Pinus* and spruce embryos [30] and increasing the embryogenic response in *Brassica* gametes [31]. Trichostatin A (TSA) and sodium butyrate (NaB) are the most widely used histone deacetylase inhibitors in plants [30,31,32,33,34,35,36,37,38].

In this work, we studied the effect of the inhibitors of histone deacetylases, NaB and TSA, on the embryogenic response of different grapevine explants derived from embryogenic cultures of the cv. Mencía [10,39]. We have also tried to better understand the role that HDAC inhibitors play in the embryogenic response in grapevine explants. With that goal, we analyzed the expression of several embryogenesis-related and HDAC-encoding grapevine genes. Since it has been suggested that the gene response to HDAC inhibitors occurs during the first hours of their application [32,40,41], we studied the expression of those genes in cotyledonary somatic embryos that were cultured for 24 h and 48 h in the presence of NaB.

## 2. Results

### 2.1. Effect of Histone Deacetylase Inhibitors on the Embryogenic Potential of Different Grapevine Explants

**Figure 1:**
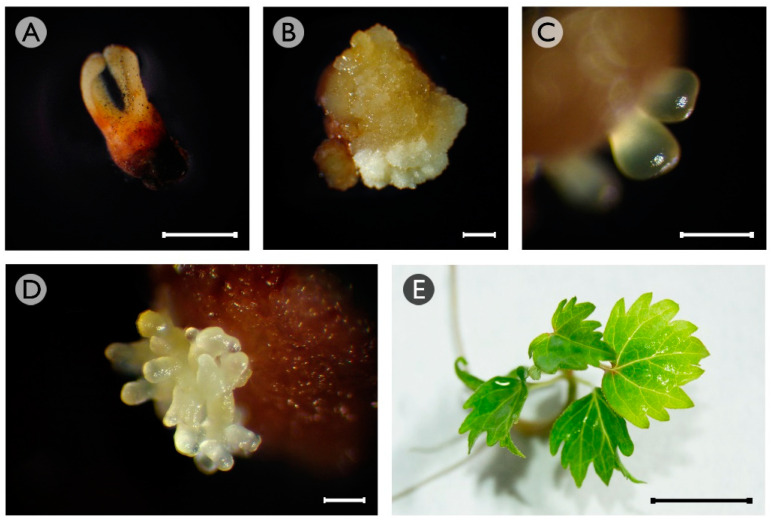
Responses observed in grapevine cv. Mencía cotyledonary somatic embryos. (**A**) Cotyledonary somatic embryo with no response and signs of necrosis after eight weeks of culture in an induction medium with 5 mM NaB. (**B**) Non-embryogenic callus originating from cotyledonary somatic embryos that were cultured in an induction medium without HDAC inhibitors for eight weeks. (**C**) Proembryos formed on the surface of cotyledonary somatic embryos that were cultured in an induction medium with 0.5 mM NaB for eight weeks. (**D**) Somatic embryo aggregate formed by secondary embryogenesis of proembryos formed from cotyledonary somatic embryos that were cultured in an induction medium with 0.5 mM NaB for eight weeks. (**E**) Grapevine microplant regenerated from somatic embryos induced in cotyledonary somatic embryos that were cultured in an induction medium with 0.5 mM NaB. Bars: 1 mm (**A**,**B**); 0.5 mm (**C**,**D**); 1 cm (**E**).

Four different explants (cotyledonary somatic embryos, recently germinated somatic embryos, and shoot apices and leaves from both in vitro-grown plants) obtained from cultures of somatic embryo aggregates of *Vitis vinifera* L. cv. Mencía, were cultured in an induction medium [39] and supplemented with different concentrations of NaB (0.5, 2 or 5 mM) or TSA (0.5, 2, or 5 μM). In all of these explants, three types of responses were observed. The first was the maintenance of their original morphology with progressive necrosis (Figure 1A). The second response was the formation of a non-embryogenic callus that was yellowish in color with some whitish areas and a watery appearance (Figure 1B). The third response was the formation of proembryos on the surface of the explants (Figure 1C), except in leaves. The formation of proembryos on the shoot apices was occasionally observed only in the presence of 0.5 mM NaB and 0.5 µM TSA (data not recorded), but these proembryos were not able to proliferate further and finally became necrotic and died. The proembryos originating from cotyledonary or recently germinated somatic embryos proliferated rapidly through secondary embryogenesis, leading to the formation of small groups of somatic embryo aggregates (Figure 1D). These aggregates developed normally using previously described procedures [10] and allowed the regeneration of plantlets without phenotypic alterations (Figure 1E).

**Figure 2:**
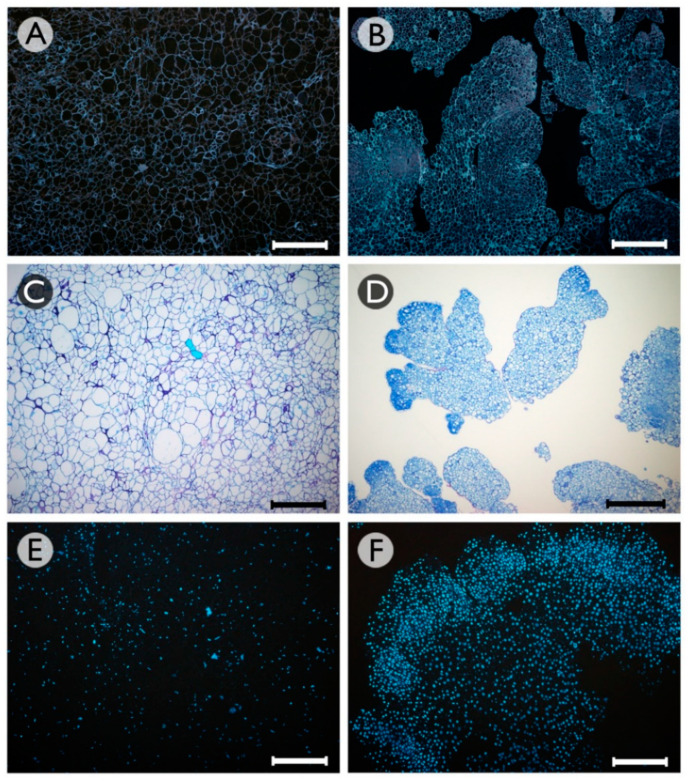
Histological analysis of non-embryogenic calli (**A**,**C**,**E**) and somatic embryo aggregates (**B**,**D**,**F**) formed from grapevine cv. Mencía cotyledonary somatic embryos that were cultured in an induction medium without HDAC inhibitors or supplemented with 0.5 mM NaB. Sections were stained with calcofluor (**A**,**B**), toluidine blue (**C**,**D**), or DAPI (**E**,**F**). Bars: 200 μm.

Histological analysis revealed that the non-embryogenic callus had an irregular structure, with cells of different sizes and shapes (Figure 2A), low proliferative activity, as revealed by their low-density cytoplasm (Figure 2C), and a low number of nuclei (Figure 2E). In contrast, the somatic embryo aggregates presented a more uniform structure, with a higher number of cells of high proliferative activity, as revealed by their smaller size (Figure 2B), dense cytoplasm (Figure 2D), and bright nuclei mainly at the periphery of those structures (Figure 2F), where the formation of proembryos could be observed (Figure 2D).

**Figure 3:**
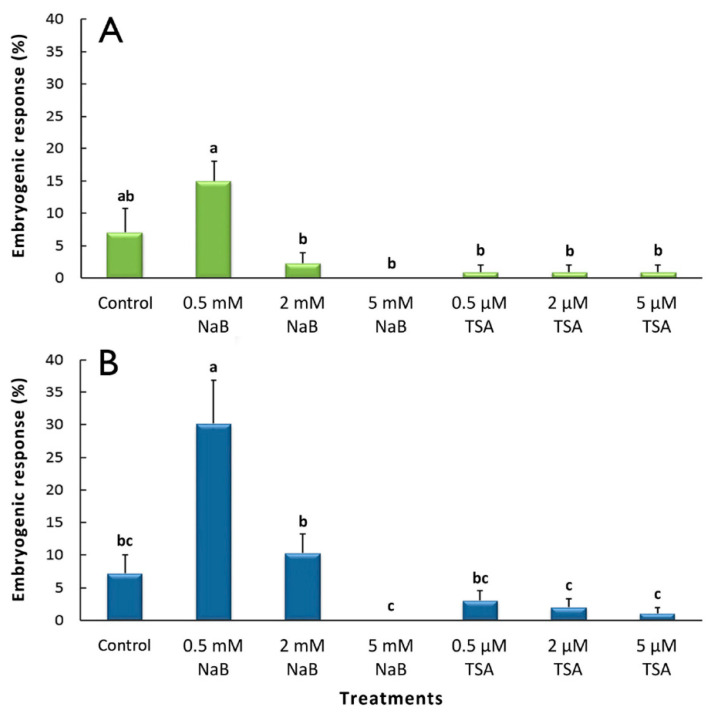
Effect of HDAC inhibitors (NaB and TSA) on embryogenic responses in grapevine cv. Mencía cotyledonary somatic embryos. The percentage of explants with an embryogenic response (mean ± standard error) after (**A**) 4 and (**B**) 8 weeks of culture is shown. Different letters indicate statistically significant differences (*p* < 0.05).

**Figure 4:**
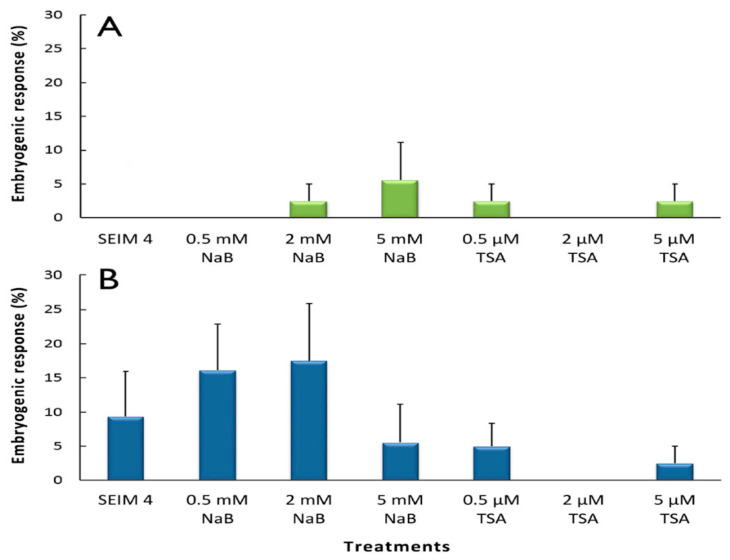
Effect of HDAC inhibitors (NaB and TSA) on the embryogenic response in grapevine cv. Mencía recently germinated somatic embryos. The percentage of explants with an embryogenic response (mean ± standard error) after (**A**) 4 and (**B**) 8 weeks of culture is shown. There were no statistically significant differences between any of the treatments (*p* < 0.05).

The effect of NaB and TSA on the embryogenic response in cotyledonary somatic embryos (Figure 3) and recently germinated (Figure 4) somatic embryos was recorded after four and eight weeks of culture. The highest percentages of embryogenic response were obtained using cotyledonary somatic embryos in which somatic embryos were newly formed on their surface after 4 weeks of culture in all media tested, except in the presence of 5 mM NaB (Figure 3A), in which no response was observed. Of all the HDAC inhibitor treatments, the best results were obtained with 0.5 mM NaB (15%), although this was not significantly different from the results obtained with the control medium. In all cases, the results obtained in the presence of TSA were very low.

The embryogenic response generally increased in cotyledonary somatic embryos after eight weeks of culture (Figure 3B) in the presence of HDAC inhibitors. The highest variation between the fourth and eighth weeks of culture was observed in the presence of 2 mM NaB, in which the embryogenic response increased from 2.4% to 10.3%. The best results were obtained again for the cotyledonary somatic embryos that were cultured in the presence of 0.5 mM NaB, with the percentage (30% on average) being significantly higher than that of the control and all other inhibitor treatments. Additionally, no response was observed in the presence of 5 mM NaB.

In the recently germinated somatic embryos, the embryogenic response after four weeks of culture (Figure 4A) was generally lower than that described above in the cotyledonary somatic embryos (Figure 3A). In this culture period, the recently germinated somatic embryos showed no embryogenic response in the control medium without inhibitors, and the best results (5.6%) were obtained in the presence of the highest NaB concentration (5 mM). In addition, an embryogenic response was also obtained with this kind of explant in the presence of 2 mM NaB, 0.5 µM TSA, and 5 µM TSA. After eight weeks of culture, the embryogenic response generally increased in the recently germinated somatic embryos (Figure 4B). The highest increase in the embryogenic response was observed in the presence of 2 mM NaB or 0.5 mM NaB. In addition, the response in the control medium without inhibitors was remarkable, although the embryos had no response after four weeks of culture (Figure 4A). No statistically significant differences between the treatments were observed in the response of the recently germinated somatic embryos.

Necrosis in the cotyledonary somatic embryos increased with time spent in the culture and due to the use of HDAC inhibitors (Table 1). In general, necrosis was more frequent in the media supplemented with NaB than with TSA and was significantly higher in the cotyledonary somatic embryos that were cultured in the presence of 2 mM NaB, both after four and eight weeks of culture. However, the use of 0.5 mM NaB, which showed the best embryogenic response in the cotyledonary somatic embryos (Figure 3), produced the lowest necrosis percentages of all the NaB treatments.

The recently germinated somatic embryos showed a lower necrosis response than cotyledonary embryos (Table 1), although the percentage of necrotic explants also increased from four to eight weeks of culture. The recently germinated somatic embryos that were cultured in the presence of 0.5 or 2 mM NaB, which showed the highest embryogenic response (Figure 4B), also showed the lowest necrosis rate after eight weeks of culture (Table 1). The most significant and highest necrosis rate in the recently germinated somatic embryos was observed after eight weeks of culture in the presence of 5 mM NaB; this necrosis rate was similar to that observed in the cotyledonary somatic embryos during the same culture period in the same medium.

### 2.2. Effect of the Histone Deacetylase Inhibitor NaB on the Expression of Somatic Embryogenesis-Related Genes and HDAC-Encoding Genes in Grapevine Cotyledonary Somatic Embryos

Due to the positive effect of the induction medium with 0.5 mM NaB on the recovery of the embryogenic responses of the cotyledonary somatic embryos (Figure 3), we studied the effect of this treatment on the expression of the genes related to embryogenic competence (*BBM*, *VvSERK1*, *VvSERK2*, *VvL1L*, *VvWUS,* and *VvWOX2*) in these explants. In addition, the expression of several genes encoding grapevine histone deacetylase enzymes (*HDT1*, *SRT1*, *SRT2*, *HDA1*, *HDA2*, *HDA3*, *HDA4*, *HDA6*, *HDA7*-9, *HDA8*, *HDA10,* and *HDA11*) was also studied to investigate whether the inhibition of histone deacetylase enzymes had any effect.

**Figure 5:**
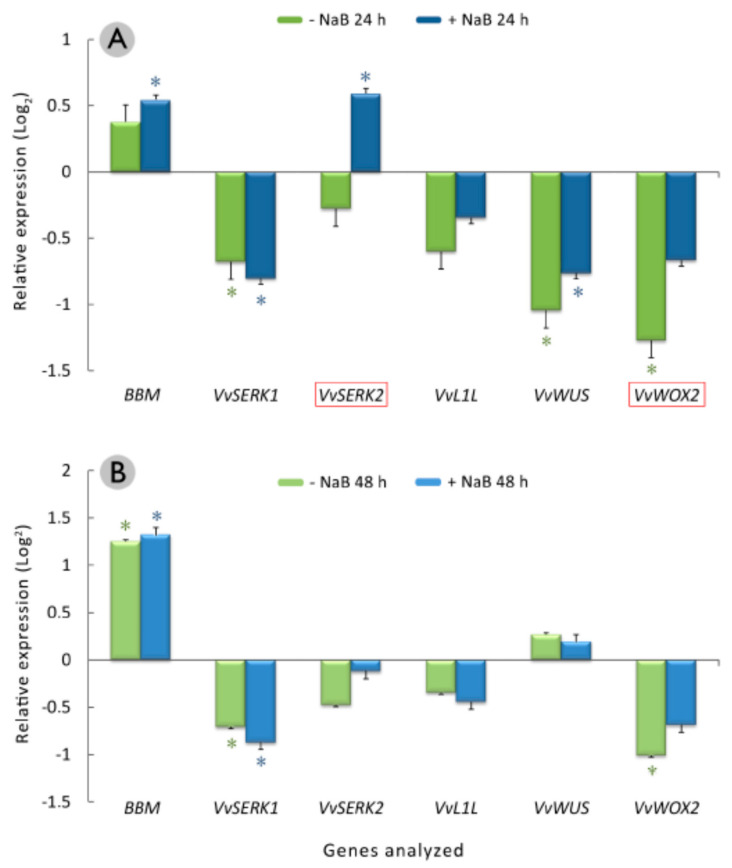
Relative expression of genes related to somatic embryogenesis in grapevine cv. Mencía cotyledonary somatic embryos that were cultured in the induction medium supplemented with 0.5 mM NaB (blue) or without NaB (green) for 24 (**A**) and 48 (**B**) h. The mean values of two independent experiments ± standard errors are shown. Asterisks indicate statistically significant differences (*p* < 0.05) between the calibrator group (cotyledonary somatic embryos used as starting material) and the analyzed group, while red squares framing genes indicate statistically significant differences (*p* < 0.05) between the NaB and no NaB treatments.

After 24 h of culture, the *BBM* and *VvSERK2* genes, related to embryogenic competence, were significantly overexpressed in the cotyledonary somatic embryos that were cultured in the presence of 0.5 mM NaB (Figure 5A). Although the expression of the *BBM* gene was not significantly different in the cotyledonary somatic embryos treated with NaB compared to those that were untreated, the expression of *VvSERK2* and *VvWOX2* was different. These two genes were significantly less underexpressed in the cotyledonary somatic embryos that were cultured in the presence of 0.5 mM NaB (Figure 5A). On the other hand, the *VvSERK1* and *VvWUS* genes were significantly underexpressed in the cotyledonary somatic embryos after 24 h of culture in the induction medium with or without 0.5 mM NaB.

After 48 h of culture, *BBM* was the only gene to be significantly overexpressed in the cotyledonary somatic embryos, with a higher relative expression level after 24 h of culture. The *VvSERK1* and *VvWOX2* genes remained significantly underexpressed. *VvWOX2* was less underexpressed in the presence of 0.5 mM NaB (Figure 5B). On the other hand, the *VvSERK2* gene was not overexpressed after 48 h in the presence of NaB, whereas the *VvWUS* gene was not underexpressed. No differences between the treatments with or without NaB were observed for any gene.

**Figure 6:**
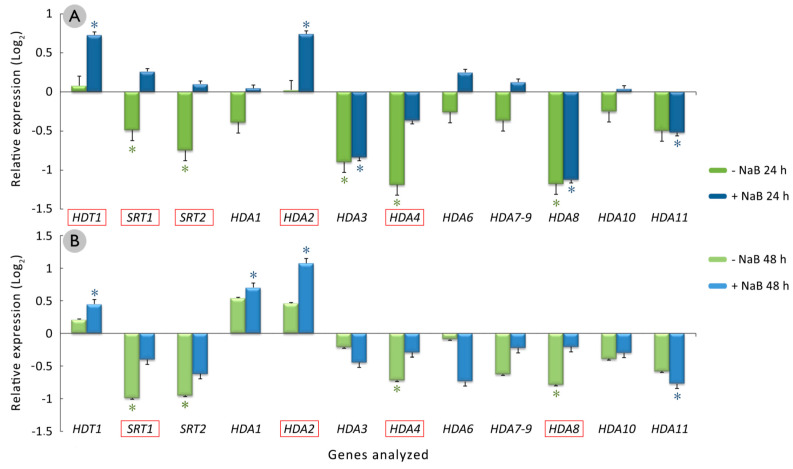
Relative expression of HDAC-encoding genes in grapevine cv. Mencía cotyledonary somatic embryos that were cultured in the induction medium supplemented with 0.5 mM NaB (blue) or without NaB (green) for (**A**) 24 and (**B**) 48 h. The mean values of the two independent experiments ± standard errors are shown. Asterisks indicate statistically significant differences (*p* < 0.05) between the calibrator group (cotyledonary somatic embryos used as starting material) and the analyzed group, while the red squares framing genes indicate statistically significant differences (*p* < 0.05) between the NaB and no NaB treatments.

The results obtained for the HDAC-encoding genes showed that after 24 h of culture of the cotyledonary somatic embryos in NaB and NaB-free media (Figure 6A), the *HDT1* and *HDA2* genes were significantly overexpressed in those embryos that were cultured in the presence of 0.5 mM NaB. In addition, these genes were differentially and significantly expressed with respect to the embryos that were cultured in the induction medium without NaB, in which their expression did not change during the first 24 h of culture.

On the other hand, the *SRT1*, *SRT2,* and *HDA4* genes were significantly underexpressed in the cotyledonary somatic embryos that were cultured for 24 h in the control medium without NaB (Figure 6A). Their expression was significantly different from that observed in the cotyledonary somatic embryos that were cultured in the presence of 0.5 mM NaB, in which their expression did not change during the first 24 h of culture. During this culture period, the *HDA3* and *HDA8* genes were significantly underexpressed in the cotyledonary somatic embryos that were cultured with or without 0.5 mM NaB, whereas the *HDA11* gene was significantly underexpressed in the embryos that were cultured only in the presence of 0.5 mM NaB.

After 48 h of culture, the number of genes whose expression was different between the treatments with or without NaB (Figure 6B) was less than that after 24 h of culture (Figure 6A). Of the genes whose expression was significantly different in the NaB treatment with respect to the control, only the *SRT1*, *HDA2*, and *HDA4* genes remained the same. In addition, the *HDA8* gene expression was significantly different between the treatments with or without NaB after 48 h of culture (Figure 6B). This is because this gene was not underexpressed in the embryos that were cultured in the presence of 0.5 mM NaB.

The *HDA1* gene was the only gene whose expression significantly changed in the cotyledonary somatic embryos after 48 h of culture in the presence of 0.5 mM NaB (Figure 6B), in which it was overexpressed. The expression did not change in the embryos that were cultured without NaB.

## 3. Discussion

Since the development of the first somatic embryogenesis protocols in grapevines [3], almost all have been based on the use of reproductive structures as explants. Less commonly, somatic embryos have also been obtained from tissues derived from vegetative structures, such as leaves and petioles [5,42,43,44,45,46], tendrils [47], or stem nodal explants [12,48]. The best induction results from vegetative tissues have been obtained from nodal segments [12,48], with efficiencies of up to 10–20% using an induction medium containing unusually high concentrations of auxins and cytokinins [12].

In this work, the highest rate of embryogenic response after 8 weeks of culture was obtained with cotyledonary somatic embryos and recently germinated somatic embryos. Although apices and leaves did not show any stable embryogenic response, the formation of proembryos was observed in shoot apices that were cultured in an induction medium supplemented with 0.5 mM NaB or 0.5 µM TSA, yet the proembryos did not proliferate and finally became necrotic. However, their formation provides the first evidence that HDAC inhibitors could be useful in the induction of embryogenesis in this type of explant.

These results confirm the importance of the degree of tissue differentiation in terms of embryogenic competence. Although it is often assumed that plant cells are totipotent, this assertion has only been possible to demonstrate in certain cells, mostly belonging to juvenile or poorly differentiated tissues [49]. Thus, the initiation of the embryogenic pathway is restricted to a small number of cells that have the potential to activate genes related to somatic embryogenesis [1]. In grapevines, this phenomenon results in the rates of the induction of somatic embryogenesis being affected by the developmental stage of the initial explants [2,3,4,5,6]. Similar results have been observed in other species; in conifers and *Arabidopsis*, embryogenic cultures are often established from zygotic embryos whose embryogenic competence is reduced when the embryos begin to germinate [30,50,51].

Treatment with NaB proved to be a very interesting alternative to increasing the embryogenic response rate in cotyledonary and recently germinated somatic embryos. Thus, the culture in the medium supplemented with 0.5 mM NaB allowed us to obtain up to 30% of the embryogenic response after 8 weeks of culture when using the cotyledonary somatic embryos as explants, a percentage that was significantly higher than that in the same medium without NaB (7.1%). In recently germinated somatic embryos, the treatments with 0.5 or 2 mM NaB also improved, although not significantly, the embryogenic response with respect to the cultures without NaB. In contrast, TSA treatments did not allow significant increases in the embryogenic response in either of these two explants.

NaB is a histone deacetylase enzyme inhibitor that has been used to increase histone acetylation levels (and thus reduce chromatin packaging) in both plant [34,35,37] and animal cells [52,53,54]. Because of the clear relationship between the degree of chromatin condensation and the embryogenic response [55,56], several researchers have conducted studies in which HDAC inhibitors were applied to observe their effect on somatic embryogenesis, with TSA being the most widely used in plant studies. Tanaka et al. [32] observed that the application of TSA induced the formation of somatic embryo-like structures in *Arabidopsis*. In *Picea* and *Pinus* germinated embryos, which have low embryogenic competence, TSA treatment was found to maintain the ability to form somatic embryos [30,57]. The exogenous application of TSA has also been used to increase the rates of haploid embryogenesis from male gametes of *Brassica napus* L. and *Arabidopsis* [31], as well as wheat [58,59].

There are no published works on the use of NaB in somatic embryogenesis, although it has been observed that the application of this compound produces an increase in histone acetylation similar to that obtained with TSA [37]. Although TSA and NaB are both HDAC enzyme inhibitors, their effect on somatic embryogenesis differed in our work; we found good results regarding the increase in the embryogenic competence of grapevine explants in media with NaB, while the TSA treatment produced worse induction rates than the media without inhibitors.

The process by which the HDAC inhibitors modify morphogenic and embryogenic processes is not clear, although several authors have noted a potential relationship between HDACs and auxins. Li et al. [31] showed that several genes related to auxin biosynthesis and transport were overexpressed in *Brassica napus* L. microspores that switched to embryogenic development from treatment with TSA. Wójcikowska et al. [60] demonstrated that somatic embryogenesis can be induced by TSA in *Arabidopsis* in the absence of auxin application, suggesting the involvement of histone acetylation in somatic embryogenic responses to auxins (reviewed in [56]).

The fact that necrosis was higher in cotyledonary somatic embryos than in recently germinated somatic embryos suggests that the latter are more tolerant to the toxic effects of the inhibitors, probably due to their larger size and more advanced stage of development. Furthermore, in the cotyledonary somatic embryos, the greatest embryogenic response was obtained with the lowest NaB concentration tested (0.5 mM), which is the NaB concentration that produced the lowest necrosis rate out of all the inhibitors combined, indicating that all the other NaB concentrations tested were excessively high. These results show the need to adjust the optimal concentration of HDAC enzyme inhibitors for somatic embryo induction, as has already been suggested by other authors using other experimental systems [61].

An expression analysis of the genes related to embryogenic competence showed that the VvSERK2 gene was significantly overexpressed in the cotyledonary somatic embryos after 24 h of culture in the presence of 0.5 mM NaB, whereas the *VvSERK1* gene was underexpressed both with and without NaB. SERK genes were first isolated in *Daucus carota* L. somatic embryos, in which they were expressed in their early stages of development [62]. Furthermore, the ectopic expression of the SERK genes has been found to increase the embryogenic potential of *Arabidopsis* [14], and they have been proposed as markers for embryogenic competence in grapevines ([63] and references therein). Our results suggest that the increase in the embryogenic response in the cotyledonary somatic embryos due to NaB treatment could be related to the significant early induction of the VvSERK2 gene, but not *VvSERK1*. In addition, Maillot et al. [63] detected a repression of both genes when they transferred the cotyledonary somatic embryos to an induction medium with 2,4-dichlorophenoxyacetic acid (2,4-D), as we detected in the cotyledonary somatic embryos that were cultured in the control medium without NaB also containing 2,4-D.

The *BBM* gene was significantly overexpressed in grapevine cotyledonary somatic embryos after 24 h of culture in an induction medium with NaB and after 48 h in both a NaB medium and the control without NaB. This overexpression could be related to the higher embryogenic response obtained with the NaB treatment. The involvement of the *BBM* gene in somatic embryogenesis was demonstrated by Boutilier et al. [15], who observed that its ectopic expression induced the formation of somatic embryos in *Arabidopsis* and *Brassica napus* L. in the absence of phytohormones. However, in *Nicotiana tabacum* L., the ectopic expression of the *BBM* genes of *Arabidopsis* and B. napus L. required the supplementation of the medium with cytokinins to induce the formation of somatic embryos [64]. As it has been determined that the *BBM* gene is inducible by auxins [65] and the induction medium contains 1 µM 2,4-D [39], the overexpression of this gene in the cotyledonary somatic embryos could be related to the presence of this phytohormone. In addition, the overexpression of the *BBM* gene in *Arabidopsis* seeds has been shown to induce the expression of the *LEC1* and *LEC2* genes, indicating that the *BBM* gene may act as a regulator of other genes related to cell identity [66].

The *VvWOX2* gene, which is one of the 12 WOX genes described in grapevines, has been associated with the early events of somatic embryogenesis [20]. In this work, the *VvWOX2* gene was significantly underexpressed in the cotyledonary somatic embryos that were cultured in the control medium without NaB, so its repression could account for the reduced rate of somatic embryogenesis in this medium. As the rate of embryogenesis increased in the cotyledonary somatic embryos that were cultured in the presence of NaB, the fact that the *VvWOX2* gene was not significantly underexpressed in grapevine cotyledonary somatic embryos after 24–48 h of culture in this medium may indicate that the acquisition of embryogenic competence is related to this gene. The activity of the *WOX *gene appears to be conserved among different species, as the same pattern of early expression is observed in *Arabidopsis* [67] and *Larix decidua* Mill. [68]. On the other hand, the *WUS * gene is also considered essential for embryogenesis and is able to promote the transition of vegetative tissue to the embryogenic state in *Arabidopsis* [16]; however, in this work, the *VvWUS* gene was significantly underexpressed in both media after 24 h but not 48 h of culture.

We observed that the expression of the *VvL1L* gene did not change in the cotyledonary somatic embryos that were cultured in media with or without NaB after 24–48 h. However, the ectopic expression of the *LEC* genes has been shown to induce the formation of somatic embryos in the absence of stress or auxins [69,70]. An analysis of the *Arabidopsis* mutants deficient in these genes showed that there is a dramatic reduction in the embryogenesis rates [71]. A possible explanation for the lack of an effect of the NaB treatment on the expression of the *VvL1L* gene could be that the expression of the LEC and L1L genes occurs at later stages of embryogenic induction. Related to this, Orłowska et al. [72] observed that in a highly embryogenic line of *Medicago truncatula* Gaertn, the expression of these genes increased significantly from the seventh day of induction. It has been reported that the expression of the *VvL1L* gene was high during the induction of grapevine somatic embryos after four weeks of culture [18], when the formation of the embryogenic structures had already been observed, whilst the expression of the *VvL1L* gene decreased during their differentiation.

An expression analysis of the HDAC-encoding genes showed that the culture of grapevine cotyledonary somatic embryos in the induction medium without NaB did not induce their expression or result in their downregulation, mostly after 24 h of culture initiation. However, treatment with 0.5 mM NaB resulted in some of the HDAC-encoding genes becoming overexpressed, even significantly, with respect to the control without NaB. These differences tended to decrease after 48 h, indicating that the gene response to NaB treatment occurs during the first hours after its application, as previously suggested [32,40,41].

The genes that showed the greatest difference in expression in the cotyledonary somatic embryos that were cultured with or without NaB after 24 h were *HDT1, SRT1, SRT2, HDA2*, and *HDA4*, with *HDA2* and HDT1 being significantly overexpressed in the NaB-treated explants with respect to those grown in the noninhibitor medium. Despite the HDA2 gene expression results found in our system, the homolog of this gene in *Arabidopsis* (*AtHDA19*) has been associated with the repression of embryogenic competence through the analysis of insertional mutants [32]. Moreover, treatment with TSA induced the expression of the AhHDA1 gene of *Arachis hypogaea* L. [40]. As these authors postulated, it may be reasonable to assume that the hyperacetylation of histones caused by the treatment with HDAC inhibitors may trigger a molecular response in these HDAC coding genes, as a mechanism for restoring normal acetylation levels.

Zhou et al. [73] reported that the induction of the *HD2* gene family, to which the *HDT1* gene of *Vitis vinifera* belongs [27], appears to be correlated with the competence of tissues to undergo somatic embryogenesis and the early stages of somatic embryo development, as the plants that transformed to overexpress a gene related to somatic embryogenesis, *BBM*, presented an accumulation of the transcripts of those genes. However, these authors could not determine whether the expression of the genes belonging to the HD2 family was due to the induction of the somatic embryos or a consequence of the overexpression of *BBM*. These results are consistent with the overexpression of the *HDT1* and *BBM* genes detected in grapevine cotyledonary embryos that were cultured in the presence of 0.5 mM NaB, which also resulted in the most significant and highest percentage of embryogenic response.

In this work, it was observed that the presence of NaB in the culture medium significantly increased the expression of the *SRT1* and *SRT2* genes with respect to the noninhibitor control medium. It should be noted that the HDAC inhibitors TSA and NaB act on type I (RPD3/HDA1) and type II (HDA) HDACs but do not affect type III HDACs (SIR2; [25]). For this reason, the differences in the expression obtained for the *SRT1* and *SRT2* genes between the two treatments (with and without NaB) cannot be due to the direct action of NaB and may involve the participation of other molecular signals in the process.

Finally, the homolog to *HDA4* in Vitis vinifera appears to be *HDA8* in *Arabidopsis*, but no literature has been found regarding the expression of this gene. Nevertheless, the overexpression of this gene after the treatment with NaB indicates that it may have a relevant role in the acquisition of embryogenic competence.

The results obtained in this work showed that treatment with NaB improved the embryogenic responses in poorly differentiated grapevine explants such as cotyledonary somatic embryos and recently germinated somatic embryos. NaB treatment was found to significantly increase the expression of the grapevine *BBM* and *VvSERK2* genes in explants that were cultured in the presence of 0.5 mM NaB after 24 h of culture, whereas the *VvWOX2* gene was underexpressed less in treated versus untreated explants. This differential expression in the genes related to embryogenic competence could explain the better embryogenic response obtained with the NaB treatment. In addition, the NaB treatment produced significant alterations in the expression of several HDAC-encoding genes, suggesting that the hyperacetylation of histones caused by this inhibitor may trigger a molecular response, possibly related to the restoration of normal acetylation levels.

## 4. Materials and Methods

### 4.1. Plant Material

**Figure 7:**
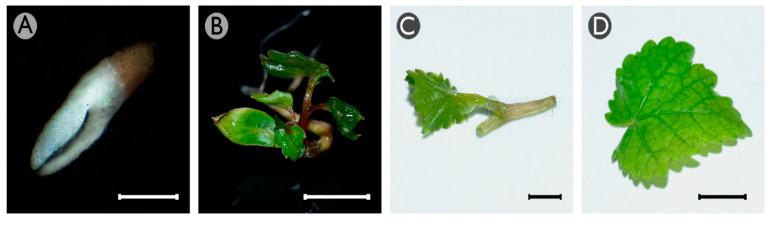
Grapevine (*Vitis vinifera* L., cv. Mencía) explants used in this work. (**A**) Somatic embryo in the cotyledonary morphological stage, (**B**) recently germinated somatic embryo, (**C**) shoot apex, and (**D**) leaf from a grapevine plant maintained in vitro. Bars: 1 mm (**A**,**C**); 1 cm (**B**); 0.5 cm (**D**).

Four different explants (cotyledonary somatic embryos, recently germinated somatic embryos, and shoot apices and leaves from both in vitro-grown plants) were used as plant materials (Figure 7). All of them were obtained from cultures of somatic embryo aggregates of *Vitis vinifera* L. cv. Mencía induced from filaments of stamens that were cultured in an induction medium [39]. The cotyledonary somatic embryos (Figure 7A) were obtained after transferring well-established somatic embryo aggregates to a semipermeable cellulose acetate membrane placed over a DM1 differentiation medium to allow their maturation [39]. The recently germinated somatic embryos (Figure 7B) were obtained by transferring cotyledonary somatic embryos to the germination medium [6]. The shoot apices (Figure 7C) and the in vitro leaves (Figure 7D) were obtained from grapevine plantlets derived from germinated somatic embryos, converted to plantlets and micropropagated as previously described [6]. The cultures in the induction or the DM1 media were maintained under continuous darkness at 24 ± 1 °C, whereas all the other cultures were maintained under a photoperiod with 16 h light with a photon flux density of 45 μmol m−^2^ s−^1^ at 24 ± 1 °C (20 ± 1 °C night temperature).

### 4.2. Effect of Treatment with Histone Deacetylase Inhibitors on the Embryogenic Potential of Different Grapevine Explants

All explants were cultured in an induction medium [39] supplemented with different concentrations of NaB (0.5, 2, or 5 mM) or TSA (0.5, 2, or 5 µM). As a control, the explants were cultured in an induction medium without the inhibitors.

Ten cotyledonary somatic embryos, four recently germinated somatic embryos, and five shoot apices or leaves were cultured in 90-mm-diameter polystyrene Petri plates containing 25 mL of medium, and at least ten plates per treatment and type of explant were used. The cultures were maintained in continuous darkness at 24 ± 1 °C for eight weeks and transferred monthly to fresh medium. The percentages of explants that produced new somatic embryos and/or showed necrosis were recorded after four and eight weeks of culture.

### 4.3. Histological Analysis

The samples of non-embryogenic callus and somatic embryo aggregates obtained after four weeks in the different media tested were fixed overnight in 4% paraformaldehyde in a phosphate buffered saline buffer (PBS) at 4 °C, dehydrated in an acetone series and embedded in Technovit 8100 resin (Heraeus Kulzer GmbH, Wehrheim, Germany). The blocks were polymerized at 4 °C overnight, and semithin sections (1 µm) were stained with 0.075% toluidine blue in water for 5 min, with 1 µg/mL 4′,6-diamidino-2-phenylindole (DAPI), and 1% Triton X-100 for 10 min in PBS buffer, or with 1% calcofluor in 0.1 M Tris-HCl buffer (pH 8.5) for 30 min. After rinsing and drying, the stained sections, except those stained with DAPI, were mounted in Eukitt (Kindler GmbH, Freiburg, Germany). The DAPI-stained sections were mounted with a 50% glycerol solution in PBS. All preparations were examined using an E800 microscope (Nikon, Tokio, Japan) equipped with a Bio-Rad MRC 1024 confocal system (Bio-Rad, Hercules, USA) and a Nikon DS-U2 digital camera.

### 4.4. Total RNA Extraction and cDNA Synthesis

Three independent samples (biological replicates) of 65 mg (fresh weight) cotyledonary somatic embryos were collected at the start and after 24 and 48 h of culture in an induction medium with and without 0.5 mM NaB. The samples were frozen with liquid nitrogen prior to total RNA extraction using the Aurum^TM^ Total RNA Mini Kit (Bio-Rad) according to the manufacturer’s instructions. The RNA concentration and purity (260/280 nm and 260/230 nm) were determined with a NanoDrop ND-1000 spectrophotometer (Thermo Fisher Scientific Inc., Waltham, MA, USA) and analyzed on an Agilent 2100 Bioanalyzer RNA 6000 Nano Lab-Chip (Agilent, Mississauga, ON, Canada) to assess the RNA quality. cDNA was synthesized from the total RNA at a ratio of 1 µg per 20 µL reaction volume using the iScript^TM^ cDNA Synthesis Kit (Bio-Rad) according to the manufacturer’s instructions, and reactions were performed on an iQ^TM^ thermal cycler (Bio-Rad).

### 4.5. Primer Design

qPCR primers (Table 2) for the analysis of the relative expression of the different tested genes were designed from the sequences of different species available either in the National Center of Biotechnology Information database or previously reported [18,19,27], except the primers used for the *VvWUS* gene, which were the primers described by Gambino et al. [20]. The qPCR primer design and optimization were performed using Gene Runner software (v3.01, Hasting Software Inc., Las Vegas, NV, USA).

### 4.6. Real-Time PCR

The relative abundance of the studied gene transcripts was determined after 24 h and 48 h of culture of the cotyledonary somatic embryos in an induction medium with or without 0.5 mM NaB. The cotyledonary somatic embryos that were collected at the beginning of the culture were considered as the calibrator group. The *Ef1-α (m)* and *GAPDH (m)* genes were used as the reference genes [39] for relative expression normalization. Three biological replicates were used per treatment, and each sample was tested in duplicate.

The gene expression analyses were performed following the Minimum Information for publication of Quantitative real-time PCR Experiments (MIQE) guidelines [74]. The qPCR reactions (20 µL), comprising 1X SsoFast^TM^ EvaGreen^®^ Supermix, Bio-Rad), 0.4 µM of each primer, and 1.66 ng cDNA, were carried out in 96-well plates in an iCycler iQ^TM^ real-time thermal cycler (Bio-Rad). The reactions were performed as follows: 1 min at 98 °C, 40 cycles of 5 s at 98 °C, and 20 s at 58 °C for annealing and extension. The dissociation curves to verify the specificity of each amplification reaction were obtained by heating the amplicons from 65 °C to 90 °C with a ramp setting at 0.5 °C/10 s. Duplicate nontemplate controls were included on each plate.

### 4.7. Data and Statistical Analysis

All experiments were repeated at least twice independently to ensure the reproducibility of the results. The data on the percentages of responses (somatic embryogenesis or necrosis) were statistically analyzed using a Mann–Whitney *U* test. Statistical tests (*p* < 0.05) were performed using PASW Statistics 18 software (IBM, New Orchard Road, New York, NY, USA). The data from the qPCR were analyzed using iCycler iQ^TM^ software (Real-Time Detection System Software, Windows ver. 3.0, Bio-Rad). The raw fluorescence data were analyzed using LinRegPCR software [75] to obtain the mean PCR efficiency for each primer pair (Table 2). The relative gene expression was determined and statistically analyzed (*p* < 0.05) using the REST-2009^©^ (Relative Expression Software Tool, ver. 2009, [76]) with PCR efficiency correction and normalization by the two reference genes indicated and compared with the 2^−ΔΔCq^ method.

## Figures and Tables

**Table 1:** Percentage of necrosis in grapevine (cv. Mencía) cotyledonary and recently germinated somatic embryos that were cultured in an induction medium supplemented with different concentrations of HDAC enzyme inhibitors (NaB and TSA) or without them (control). Means and standard error are shown. Different letters indicate statistically significant differences (*p* < 0.05).

Inhibitor Treatments	Cotyledonary Somatic Embryos		Recently Germinated Somatic Embryos	
	Necrosis (%) after 4 Weeks	Necrosis (%) after 8 Weeks	Necrosis (%) after 4 Weeks	Necrosis (%) after 8 Weeks
Control	7.50 ± 3.66 a	16.07 ± 8.38 a	0.00 ± 0.00 a	27.50 ± 11.06 a
0.5 mM NaB	26.00 ± 9.09 ab	40.00 ± 9.89 ab	0.00 ± 0.00 a	0.00 ± 0.00 a
2 mM NaB	72.72 ± 8.07 c	83.83 ± 6.05 c	0.00 ± 0.00 a	5.00 ± 3.33 a
5 mM NaB	38.89 ± 5.79 b	55.08 ± 1.89 b	16.67 ± 9.32 a	58.33 ± 11.79 b
0.5 µM TSA	2.00 ± 1.33 a	29.00 ± 6.79 ab	7.50 ± 5.34 a	20.00 ± 7.26 a
2 µM TSA	7.00 ± 2.60 a	29.00 ± 6.74 ab	7.50 ± 5.34 a	17.50 ± 9.17 a
5 µM TSA	25.00 ± 7.03 ab	56.00 ± 7.48 b	17.50 ± 3.82 a	25.00 ± 6.45 a

**Table 2:** Nucleotide sequence, amplicon size and qPCR efficiency of the oligonucleotide primers used in this study. We designed all primers except the qPCR primer pair for the Vv*WUS * gene [20].

Gene	Accession No.	Primer Sequences (Forward/Reverse)	Amplicon Size (bp)	qPCR Efficiency
*VvSERK1*	VIT_18s0164g00070	TCAGAAGTGGTGAGAATGCTG/ GTCCACAATCCAATCAGAGTTG	126	1.90
*VvSERK2*	VIT_07s0031g01410	CGAGGTTGTCCGAATGCTT/ ACGATCCATTCAGAACACCG	120	1.89
*VvL1L*	VIT_00s0956g00020	CTGTGAGGGAACAAGACAGG/ GCATCATCCGAGATTTTGGC	94	1.94
*VvWUS*	VIT_04s0023g03310	CCCATGCACGCTGAGGAT/ CGGATTCGGGCTTAATGTTG	52	1.94
*VvWOX2*	XM_002281125.3	CCTTTGTTCCCTCCTCCATG/ AAAAGCACCTTGGGGTACTG	98	1.92
*VvBBM*	VIT_04s0023g00950	GTGACCAGACACCATCAGCAT/ ATCCTCGAAACTTAATGGCAG	142	1.91
*HDT1*	VIT_08s0007g03940	CTGTGGATAATGGGAAGCCTC/ ACGATCTTCACCTGCTTAGC	76	1.99
*SRT1*	VIT_19s0015g00570	ATTTCAAGGTTTCGACAGACTGTTT/ GATCTGGGATGGGCTTTTTCT	129	1.89
*SRT2*	VIT_07s0031g02510	TGGTATTGACTGGAGCTGGA/ AACGTACAAACTCCTGATGGG	114	1.91
*HDA1*	VIT_14s0006g01820	GCCCTTTAGCCCATCATCAC/ CTCTGTGTGCCTTGAACTCA	144	1.93
*HDA2*	VIT_03s0038g04240	GCTGATTTTGGAACCACAACC/ TTTTTCACCTCAGAAGCCACTC	120	1.89
*HDA3*	VIT_17s0000g04120	AGGCTTTAATGGACAGCATGA/ TCTTCCCGACAATTTTCATCAGA	81	1.98
*HDA4*	VIT_06s0080g00210	GAACGGGAGATCGGGGATAT/ CCATTCGGATCAAAAGCACTT	124	1.85
*HDA6*	VIT_17s0000g09070	AGACCTAAACCTCGCATTTGG/ CCAGTGACACCCCTCATCTC	131	1.90
HDA 7-9 *	VIT_06s0061g01510	AACTTAAATAGCAAATCGTATATTGGAAC/ AACCTCTTGCATCTGTACGC	99	1.95
*HDA8*	VIT_17s0000g07280	CATTCGAGTCAACATGGCGT/ TCTTCAGAATCAGAGCTTGCG	148	1.84
*HDA10*	VIT_04s0008g00910	GTTGAAGTAGTGAGTGGGACC/ AGGATCAAACATGCGTCCAG	87	1.94
*HDA11*	VIT_04s0044g01510	GGTGAAGGAGCGACACTAAA/ CCCATCATAACCAGCTGAGAC	141	1.86

* HDA7 and HDA9 represent the same duplicated sequence on grapevine chromosomes 15 and 6, respectively [27].

## Data Availability

Not applicable.

## Abbreviations

2:4-D-2:4-dichlorophenoxyacetic acid, DAPI-4′,6-diamidino-2-phenylindole, HAT-histone acetyltransferase, HDAC-histone deacetyltransferase, PBS-phosphate-buffered saline, TSA-trichostatin A, and NaB-sodium butyrate.
